# Bioconvective flow of Maxwell nanoparticles with variable thermal conductivity and convective boundary conditions

**DOI:** 10.1038/s41598-023-51113-0

**Published:** 2024-01-17

**Authors:** Hassan Hanafy, Iskander Tlili

**Affiliations:** 1 Department of Physics, College of Science, Al-Zulfi, Majmaah University, 11952 Al-Majmaah, Saudi Arabia; 2 Physics Department, Faculty of Sciences, Beni-Suef University, Beni Suef, 62514 Egypt

**Keywords:** Mathematics and computing, Nanoscience and technology

## Abstract

Owing to recent development in the thermal sciences, scientists are focusing towards the wide applications of nanofluids in industrial systems, engineering processes, medical sciences, enhancing the transport sources, energy production etc. In various available studies on nanomaterials, the thermal significance of nanoparticles has been presented in view of constant thermal conductivity and fluid viscosity. However, exponents verify that in many industrial and engineering process, the fluid viscosity and thermal conductivity cannot be treated as a constant. The motivation of current research is to investigates the improved thermal aspects of magnetized Maxwell nanofluid attaining the variable viscosity and thermal conductivity. The nanofluid referred to the suspension of microorganisms to ensure the stability. The insight of heat transfer is predicted under the assumptions of radiated phenomenon. Additionally, the variable thermal conductivity assumptions are encountered to examine the transport phenomenon. Whole investigation is supported with key contribution of convective-Nield boundary conditions. In order to evaluating the numerical computations of problem, a famous shooting technique is utilized. After ensuring the validity of solution, physical assessment of problem is focused. It is claimed that velocity profile boosted due to variable viscosity parameter. A reduction in temperature profile is noted due to thermal relaxation constant.

## Introduction

In recent developments in nanoscience’s, the attention of researchers increases in study of nanofluids to suggests more valuables applications. Basically, nanofluids reports the decomposition of tiny metal particles with base materials. The fundamental aspects of nanomaterials are higher thermal capacitance and stable heating aspect. Diverse applications in modern engineering and technological systems is discussed for nanofluids in extrusion systems, engine of vehicles, nuclear reactions, cooling systems etc. In the modern medical sciences, scientists have contributed the applications of tiny particles in the cancer treatment and chemo-therapy. Choi^1^ initiated the basic research on nanoparticles with support of novel experimental results. Kumar et al.^2^ deduced the nanofluid role in enhancing heat transfer for non-Newtonian slip flow. Santos et al.^3^ preserved the statistical analysis regarding nanofluid problem and suggested improved thermal performances. Sohail et al.^4^ tested the Sutterby nanofluid conveying stretching cylinder via modified laws. Muhammad et al.^5^ reported the hybrid class of nanofluid to boosted the quantitative impact of base liquid. Babazadeh et al.^6^ discussed the shape factors for enclosure filled with nanoparticles in porous space. The lubricated constraints for nanofluid under the melting heating source was focused by Alqarni et al.^7^. Saeed et al.^8^ examined the Darcy Forchheimer analysis for nanofluid in porous moving frame. Hamrelaine analyzed the convergent/divergent channel flow analysis subject to nanofluid. The esterification of nanofluid based on pressure driven phenomenon in circular surface have been evaluated by Shahzad et al.^10^. Acharya^11^ observed the entropy generation aspects of copper nanofluid for natural convective flow. Sajid et al.^12^ discussed the Reiner-Philippoff hybrid nanofluid with quadratic chemical analysis. The aluminum nanoparticles thermal attention was tested by Hanif et al.^13^. Iqbal et al.^14^ justified the role of Lorentz force while incorporating nanofluid thermal prediction with slip features. Mabood et al.^15^ organized the Wu’s slip impact for nanofluid containing the variable thermal conductivity. Chu et al.^16^ addressed the chemical reactive analysis for nanofluid via computational model.

The microorganisms floating due to lower densities causes the phenomenon of bioconvection. The suspension of microorganisms in upper regime is associated to the instability of uniform structure. The uniform movement of nanoparticles does not effect the suspension of microorganisms and signifies applications in biotechnology and bio-engineering. Alwatban et al.^17^ addressed the bioconvection outcomes in Eyring-Powell nanofluid. Khan et al.^18^ deduces bioconvective observations while defining the heating onset of thixotropic nanofluid. Khan et al.^19^ analyzed the entropy generation framework in suspension of microorganisms in nanofluid. Bafakeeh et al.^20^ examined the bioconvective onset in viscoelastic nanofluid. The features of activation energy in bioconvective flow was reported by Khan et al.^21^. Bhatti et al.^22^ announced the higher order slip mechanism in nanofluid flow with microorganisms. Irfan^23^ investigated the Joule heating impact for Maxwell nanofluid. Ali et al.^24^ analyzed the joint rheology of Casson-Williamson nanofluid with significance of thermos-diffusion phenomenon. The nonlinear mixed convection flow associated to Carreau nanofluid was contributed by Irfan^25^.

Continuous research in non-Newtonian materials is focused by investigators in recent years due to their prestigious applications in industrial systems and manufacturing processes. The novelty of non-Newtonian materials is dynamic and relatively complex. In order to evaluate the rheological aspects of nonlinear materials, difference non-Newtonian models are prosed in literature. In such categories, Maxwell fluid is one which occupies the interesting relaxation time features. Maxwell fluid model is important in manufacturing processes and cosmetics. Many studies are available which reports the rheology of Maxwell fluid. Some research on Maxwell nanofluid is seen in Refs.^27–29^.

The aim of current research is to present the bioconvective flow of Maxwell nanofluid with in presence of thermal radiation and external heat source. The inspection of transport phenomenon is predicted under the assumptions of variable viscosity and variable thermal conductivity. Furthermore, analysis is subject to the activation energy. The convective-Nield thermal conditions are imposed to analyze the flow. The convective-Nield thermal constraints are associated to the consideration of convective conditions for heat and mass transfer phenomenon. The computations of problem are performed via shooting technique. Current investigation presents the answer to following research questions:

- How velocity profile of Maxwell nanofluid truncated for variable viscosity?
- What is the significance of temperature dependent thermal conductivity for enhancing the heat transfer rate?
- Why external heat source and radiative phenomenon is important to inspect the heat transfer rate?
- What is the contribution of bioconvection phenomenon in nanofluid flow?
- How does heat and mass transfer pattern fluctuated with interaction of convective-Nield thermal constraints?

## Physical description and formulation of problem

**Figure 1:**
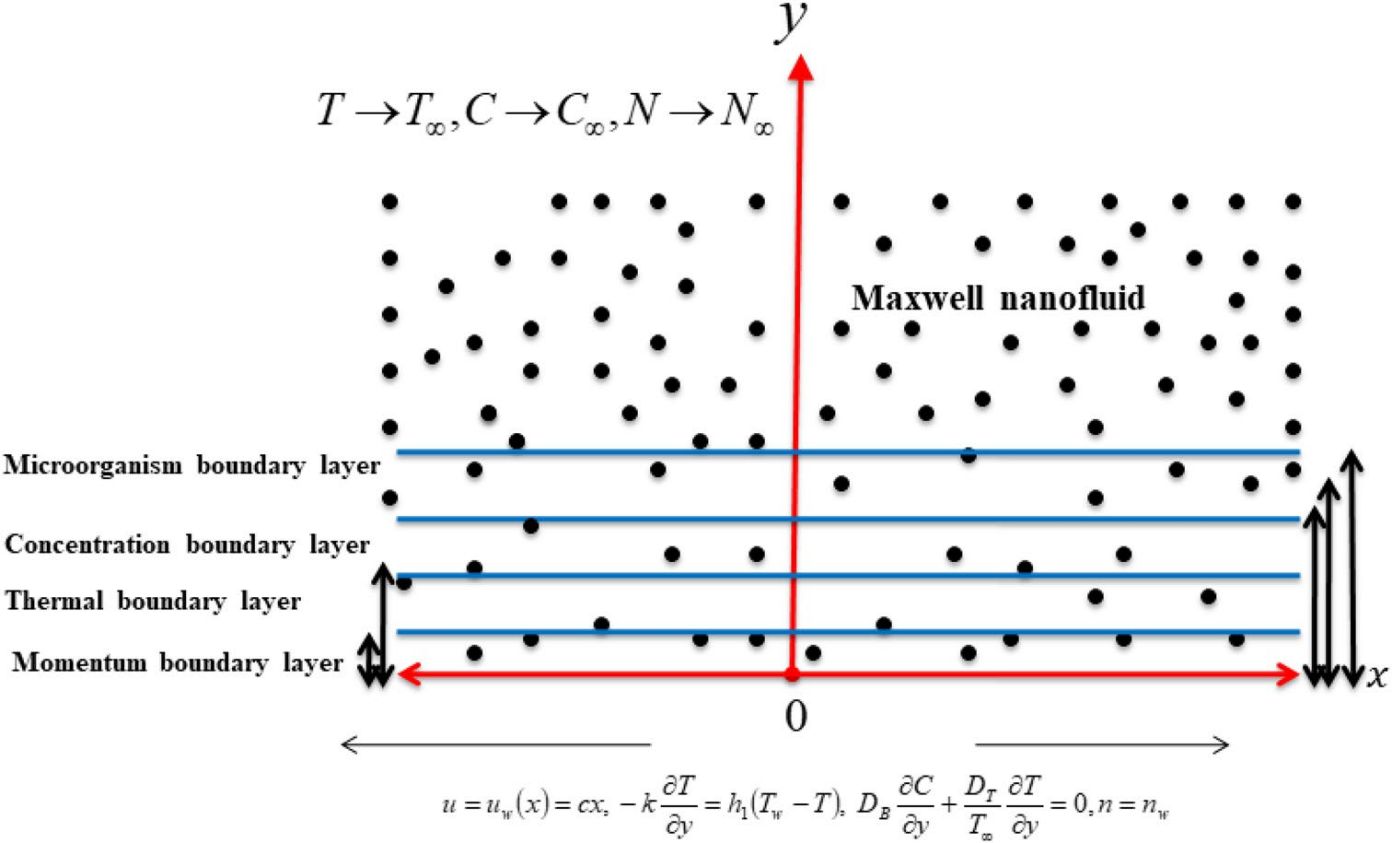
Flow geometry of problem.

Let us assumed a two-dimensional and steady transport of Maxwell nanofluid with suspension of microorganisms over stretched surface. The normal direction magnetic force is implemented. The variable consideration of viscosity is taken into contributed. The Cartesian system is used for modeling and configuration. The velocity components $u$ and $v$ are taken in horizontal and normal directions, respectively. Flow configuration is presented in Fig. 1. In concentration equation, the activation energy outcomes are utilized. The modification in energy equation is supported with radiative phenomenon and external heat source. Defining temperature, surface temperature, free stream temperature, concentration, and ambient concentration is defined as $T,T_{w} ,T_{\infty } ,C$ and $C_{\infty }$, respectively. Moreover, $n$, $n_{w}$ and $n_{\infty }$ be motile density, surface motile density and free stream motile density, respectively. Under these assumptions, the flow model is illustrated with help of following equations^28,31^:

1 $$\frac{\partial u}{{\partial x}} + \frac{\partial v}{{\partial y}} = 0,$$

2 $$u\frac{\partial u}{{\partial x}} + v\frac{\partial v}{{\partial y}} + \lambda \left( {u^{2} \frac{{\partial^{2} u}}{{\partial x^{2} }} + v^{2} \frac{{\partial^{2} u}}{{\partial y^{2} }} + 2uv\frac{{\partial^{2} u}}{\partial x\partial y}} \right) = \frac{1}{{\rho_{f} }}\frac{\partial }{\partial y}\left( {\mathop \mu \limits^{ \simeq } (T)\frac{\partial u}{{\partial y}}} \right) - \frac{{\sigma B_{o}^{2} }}{{\rho_{f} }}\left( {u + \lambda v\frac{\partial u}{{\partial y}}} \right),$$

3 $$u\frac{\partial T}{{\partial x}} + v\frac{\partial T}{{\partial y}} = \frac{1}{{\rho c_{p} }}\frac{\partial }{\partial y}\left( {\mathop K\limits^{ \simeq } (T)\frac{\partial T}{{\partial y}}} \right) - \frac{1}{{\rho c_{p} }}\frac{{\partial q_{r} }}{\partial y} + \frac{{Q_{0} }}{{\rho c_{p} }}\left( {T - T_{\infty } } \right)\, + \tau \left\{ {D_{B} \left( {\frac{\partial C}{{\partial y}}\frac{\partial T}{{\partial y}}} \right) + \left( {\frac{{D_{T} }}{{T_{\infty } }}} \right)\,\left( {\frac{\partial T}{{\partial y}}} \right)^{2} } \right\},$$

4 $$u\frac{\partial C}{{\partial x}} + v\frac{\partial C}{{\partial y}} = D_{B} \frac{{\partial^{2} C}}{{\partial y^{2} }} + \left( {\frac{{D_{T} }}{{T_{\infty } }}} \right)\,\frac{{\partial^{2} T}}{{\partial y^{2} }} - kr^{2} \left( {C - C_{\infty } } \right)\left( {\frac{T}{{T_{\infty }^{n} }}} \right)\exp \left( { - \frac{{E_{a} }}{{k_{1} T}}} \right).$$

5 $$u\frac{\partial n}{{\partial x}} + v\frac{\partial n}{{\partial y}} + \frac{{\hat{b}\hat{w}}}{{\left( {C_{w} - C_{\infty } } \right)}}\left[ {\frac{\partial }{\partial y}\left( {n\frac{\partial C}{{\partial y}}} \right)} \right] = D_{m} \frac{{\partial^{2} n}}{{\partial y^{2} }},$$

The appropriate conditions are

6 $$u = u_{w} \left( x \right) = cx,\, \, v = 0,\, \, - k\frac{\partial T}{{\partial y}} = h_{1} \left( {T_{w} - T} \right),\, \, D_{B} \frac{\partial C}{{\partial y}} + \frac{{D_{T} }}{{T_{\infty } }}\frac{\partial T}{{\partial y}} = 0,n = n_{w} {\text{ at }}y = 0$$

7 $$u \to 0,T \to T_{\infty } ,\,C \to C_{\infty } {,}\,n \to n_{\infty } {\text{, when }}y \to \infty .$$

with physical quantities like relaxation time $\lambda$, fluid density $\rho_{f}$, temperature dependent viscosity $\mathop \mu \limits^{ \simeq } (T)$, electrical conductivity $\sigma$, magnetic field strength $B_{0}$, specific heat $\rho c_{p}$, variable thermal conductivity $\mathop K\limits^{ \simeq } (T)$, radiative flux $q_{r}$, heat source coefficient $Q_{0}$, Brownian diffusion $D_{B}$, chemotaxis constant $\hat{b}$, mean absorption coefficient $k^{ * }$ and microorganisms diffusion constant $D_{m}$.

Defining the radiative flux:

8 $$\frac{{\partial q_{r} }}{\partial y} = - \frac{{16\sigma^{ * } T_{\infty }^{3} }}{{3K^{ * } }}\frac{{\partial^{2} T}}{{\partial y^{2} }},$$

where $\sigma^{ * }$ and $K^{ * }$ are Stefan Boltzmann constant and mean absorption coefficient. The expression of viscosity is^18^

9 $$\mathop \mu \limits^{ \simeq } (T) = \mathop {\mu_{o} }\limits^{ \simeq } \exp \left[ { - a(T - T_{\infty } )} \right].$$

Now thermal conductivity is:

10 $$\mathop K\limits^{ \simeq } (T) = K\exp \varepsilon \left( {\frac{{T - T_{\infty } }}{{T_{w} - T_{\infty } }}} \right).$$

Let us define new variables:

11 $$\eta = \sqrt{\frac{c}{v}} y,\,\psi = \sqrt {c\nu } xf\left( \eta \right),\, \, \theta \left( \eta \right) = \frac{{T - T_{\infty } }}{{T_{w} - T_{\infty } }},\, \, \varphi \left( \eta \right) = \frac{{C - C_{\infty } }}{{C_{w} - C_{\infty } }},\chi \left( \eta \right) = \frac{{n - n_{\infty } }}{{n_{w} - n_{\infty } }}$$

$$u = \frac{\partial \psi }{{\partial y}} = cxf^{\prime}\left( \eta \right),\,v = - \frac{\partial \psi }{{\partial x}} = \sqrt {cv} f\left( \eta \right).$$

The resulting dimensionless system is:

12 $$\left( {1 - \gamma_{1} \theta } \right)\,\left[ {f^{\prime\prime\prime} - \gamma_{1} \theta^{\prime}f^{\prime\prime}} \right] - \beta \left( {\left( f \right)^{2} f^{\prime\prime\prime} - 2ff^{\prime}f^{\prime\prime}} \right) - \left( {f^{\prime}} \right)^{2} + ff^{\prime\prime} = M\left( {f^{\prime} - \beta ff^{\prime\prime}} \right),$$

13 $$\left( {1 + \in \theta + \frac{4}{3}R_{d} } \right)\,\theta^{\prime\prime} = - \in \left( {\theta^{\prime}} \right)^{2} - \mathop {\Pr }\limits \left( {f\theta^{\prime} + Q\theta + N_{b} \theta^{\prime}\varphi^{\prime} + N_{t} \left( {\theta^{\prime}} \right)^{2} } \right),$$

14 $$\phi^{\prime\prime} + Le\Pr f\varphi^{\prime} + \frac{{N_{t} }}{{N_{b} }}\theta^{\prime\prime} - \Pr Le\left( {1 + \delta \theta } \right)^{n} \exp \left( {\frac{ - E}{{1 + \delta \theta }}} \right)\phi = 0,$$

15 $$\chi^{\prime\prime} + Lbf\chi^{\prime} - Pe\left[ {\phi^{\prime\prime}\left( {\chi + \delta_{1} } \right) + \chi^{\prime}\phi^{\prime}} \right] = 0,$$

with:

16 $$\left. \begin{aligned} & f(0) = 0,\, \, f^{\prime}(0) = 1,\, \, f^{\prime}(\infty ) \to 0,\, \\ & \theta^{\prime}(0) = - Bi\left( {1 - \theta \left( 0 \right)} \right),\, \, \theta (\infty ) \to 0,\, \\ & N_{b} \varphi^{\prime}(0) + N_{t} \theta^{\prime}(0) = 0,\, \, \varphi (\infty ) \to 0. \\ & \chi \left( 0 \right) = 1,\chi^{{^{\prime } }} (\infty ) \to 0. \\ \end{aligned} \right\}$$

In above mathematical expression, $\Pr$ the Prandtl number, $\gamma_{1}$ the Biot number, $R_{d}$ the radiation parameter, $Le$ the Lewis number, $\beta$ the Maxwell parameter, $M$ Hartmann parameter, $N_{b}$ the Brownian motion parameter, $N_{t}$ the thermophoresis parameter, $\varepsilon$ the curie temperature parameter and $Q$ the heat source/sink parameter, microorganism difference parameter $\delta_{1}$ Peclet number $Pe$

$$\begin{gathered} \mathop {\Pr }\limits = \frac{{\mu c_{p} }}{k},\, \, \gamma_{1} = c\left( {T_{w} - T_{\infty } } \right),\, \, Rd = \frac{{4\sigma^{ * } T_{\infty }^{3} }}{{3K^{ * } K}},\, \, Le = \frac{v}{{D_{B} }}{, }M = \frac{{\sigma B_{o}^{2} }}{{\rho_{f} c,}} \hfill \\ \, \beta = \lambda c,\, \, N_{b} = \frac{{\tau D_{B} \left( {C_{w} - C_{\infty } } \right)}}{\nu },\,N_{t} = \frac{{\tau \left( {T_{w} - T_{\infty } } \right)}}{{\nu T_{\infty } }},\, \, \varepsilon = \frac{{T_{\infty } }}{{T_{w} - T_{\infty } }},\, \, Q = \frac{{Q_{0} }}{{\rho c_{p} }}. \hfill \\ \end{gathered}$$

$$\delta_{1} = \frac{{N_{\infty } }}{{N_{w} - N_{\infty } }},\,\,Pe = \frac{{\hat{b}\hat{w}}}{{D_{m} }}$$

The dimensionless skin friction coefficient $C_{fx}$, local Nusselt number $Nu_{x}$ and the local Sherwood number $Sh_{x}$, motile density number $Nn_{x}$ is:

17 $$\begin{gathered} (Re_{x} )^{1/2} C_{{f_{x} }} = (1 - \gamma_{1} \theta (0))f^{\prime\prime}(0),\,\; \hfill \\ (Re_{x} )^{ - 1/2} Nu_{x} = - \left( {1 + \frac{4}{3}Rd} \right)\,\theta^{\prime}(0), \hfill \\ (Re_{x} )^{ - 1/2} Sh_{x} = - \varphi^{\prime}\left( 0 \right), \hfill \\ (Re_{x} )^{ - 1/2} Nn_{x} = - \chi^{\prime}\left( 0 \right), \hfill \\ \end{gathered}$$

where $Re_{x} = U_{w} (x)x/\nu$ is local Reynolds number.

## Computational procedure

The numerical computations are carried out for tracking the solution of desired formulated systems. The shooting technique is implemented to perform the approximate solution. The motivations for utilizing the numerical scheme is due to excellent accuracy. The residual error associated to this scheme is also very convincing. Moreover, this scheme does not involve any complicated discretization steps like other numerical algorithms. For this purpose, the initial value system is obtained. The simulations are carried out under following assumptions:

18 $$\begin{gathered} f = y_{1} ,f^{\prime} = y_{2} ,f^{\prime\prime} = y_{3} ,f^{\prime\prime\prime} = y^{\prime}_{3} ,\theta = y_{4} ,\theta^{\prime} = y_{5} ,\theta^{\prime\prime} = y^{\prime}_{5} , \hfill \\ \phi = y_{6} ,\phi^{\prime} = y_{7} ,\phi^{\prime\prime} = y^{\prime}_{7} ,\chi = y_{8} ,\chi^{\prime} = y_{9} ,\chi^{\prime\prime} = y^{\prime}_{9} \hfill \\ \end{gathered}$$

19 $$y_{3}^{\prime } = \left( {\tfrac{1}{{1 - \gamma_{1} y_{4} - \beta y_{1}^{2} }}} \right)\,\left[ {\gamma_{1} \left( {1 - \gamma_{1} y_{4} } \right)\,y_{3} y_{5} + y_{2}^{2} - y_{1} y_{3} - 2\beta y_{1} y_{2} y_{3} + My_{1} - \beta My_{1} y_{3} } \right]$$

20 $$y_{5}^{\prime } = \left( {\tfrac{ - 1}{{1 + \varepsilon y_{4} - \tfrac{4}{3}\mathop {R_{d} }\limits^{?} }}} \right)\,\left[ {\varepsilon y_{5}^{2} + \Pr \left( {y_{1} y_{5} + Qy_{4} + N_{b} y_{5} y_{7} + N_{t} y_{5}^{2} } \right)} \right]$$

21 $$y_{7}^{\prime } = - \Pr Ley_{1} y_{7} - \tfrac{{N_{t} }}{{N_{b} }}yy_{2} - \Pr (Le)\left( {1 + \delta y_{4} } \right)\exp \left( {\left( {\frac{ - E}{{1 + \delta y_{4} }}} \right)y_{6} } \right)$$

22 $$y_{9}^{\prime } = - \left( {Lbfy_{9} - Pe\left[ {y_{5}^{\prime } \left( {y_{8} + \delta_{1} } \right) + y_{9} y_{7} } \right]} \right),$$

along with initial conditions

23 $$\left. \begin{aligned} & y_{1} (0) = 0,\, \, y_{2} (0) = 1,\, \, y_{2} (\infty ) \to 0,\, \\ & y_{5} (0) = - Bi\left( {1 - y_{5} \left( 0 \right)} \right),\, \, y_{4} (\infty ) \to 0,\, \\ & Nby_{7} \left( 0 \right) + Nty_{4} \left( 0 \right) = 0,\, \, y_{7} (\infty ) \to 0. \\ & y_{8} \left( 0 \right) = 1,y_{8}^{{^{\prime } }} (\infty ) \to 0. \\ \end{aligned} \right\}$$

## Validation of results

The results are validated and verified in Table 1. The comparative analysis is worked out under limiting case with studies of Chen^29^ and Iqbal et al.^14^. A fine accuracy of current results is noted with these available investigations.

**Table 1:** Comparative analysis for $- \theta^{\prime}(0)$ with Chen^26^ and Iqbal et al.^14^.

$\Pr$	Chen^29^	Iqbal et al.^14^	Present results
0.72	0.46315	0.46315	0.46315
1.0	0.58199	0.58199	0.58199
3.0	1.16523	1.16523	1.16522
7.0	1.89537	1.89537	1.89538

## Results and discussion

**Figure 2:**
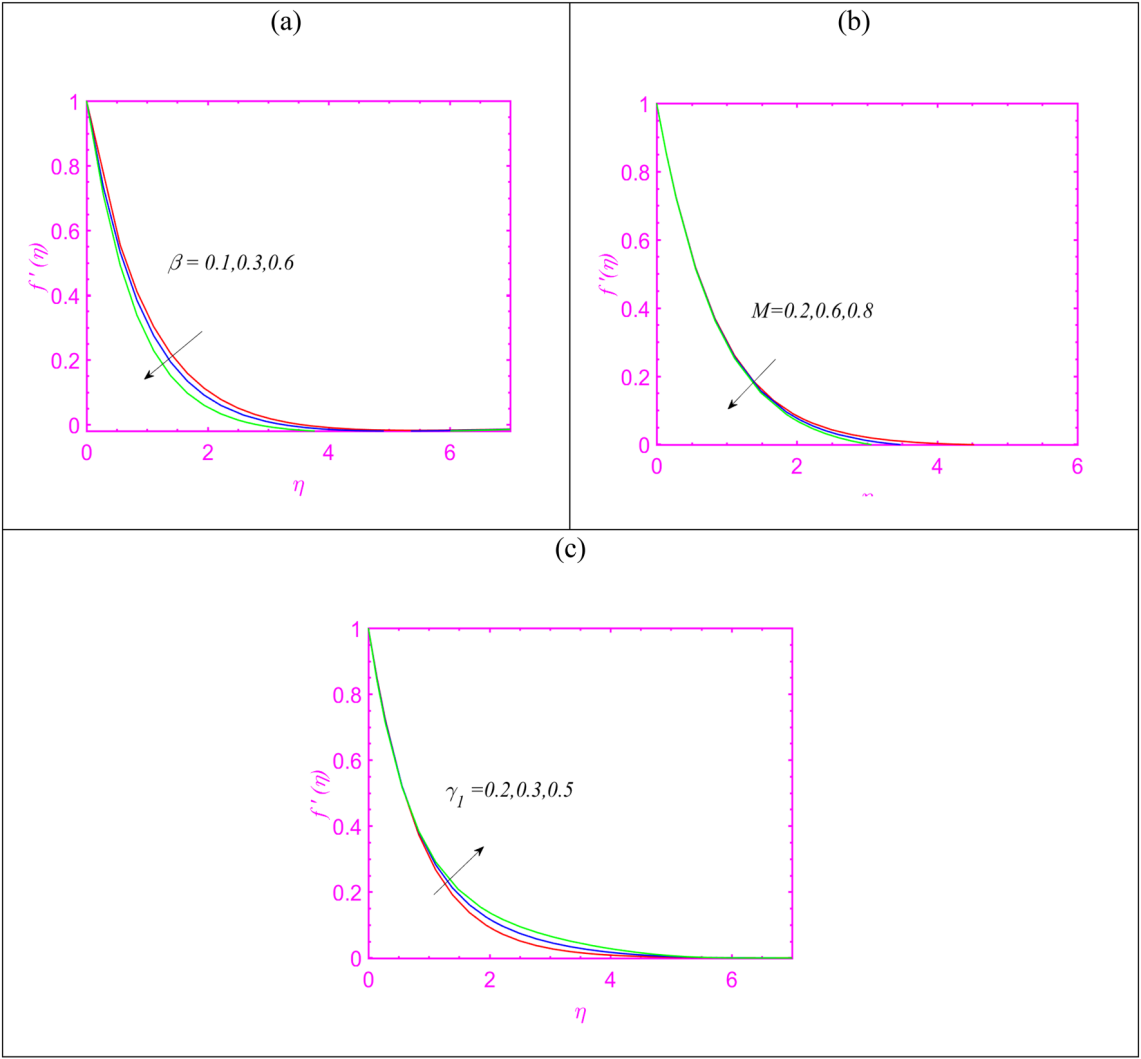
(**a**–**c**) Change in velocity for (**a**) $\beta$, (**b**) $M$ and (**c**) $\gamma_{1}$.

**Figure 3:**
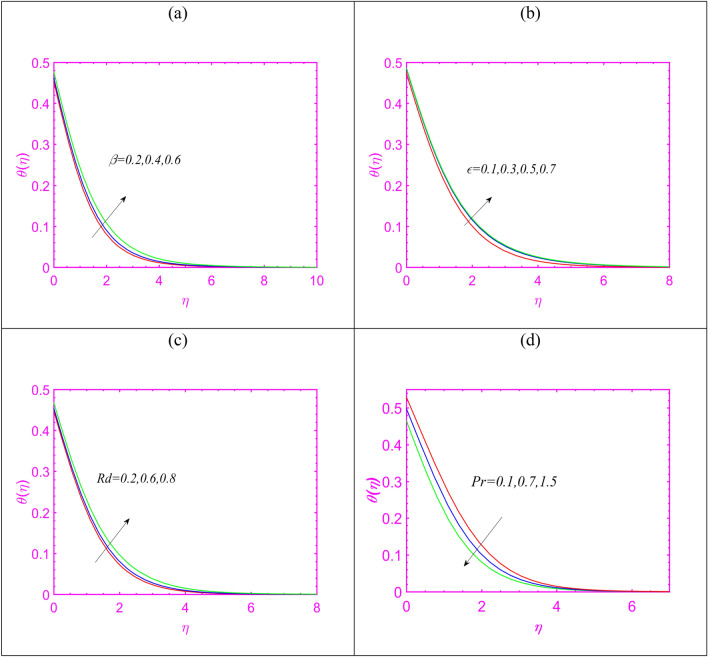
(**a**–**d**) Assessment of temperature for (**a**) $\beta$, (**b**) $\varepsilon$, (**c**) $Rd$ and (**d**) $\Pr .$

Now, the physical aspects of parameters in given flow problem is observed. For this purpose, a graphical analysis is prepared to investigate the thermal phenomenon. In order to analyze the graphical results, the numerical values assigned to parameters are $\Pr = 0.5,$ $\gamma_{1} = 0.2,$ $R_{d} = 0.5,$ $Le = 0.3,$ $\beta = 0.5,$ $M = 0.4,$ $N_{b} = 0.3,$, $N_{t} = 0.3,$ $\varepsilon = 0.2,$ $Q = 0.4$, $\delta_{1} = 0.2$ and $Pe = 0.5.$ Figure 2a preserves observations for velocity $f^{\prime}$ by utilizing the values of $\beta$. A slower velocity impact is observed when $\beta$ attributed maximum values. Such control in velocity is due to property of relaxation time as fluid takes time to attains its original position. Figure 2b pronouncing the predication in $f^{\prime}$ upon enriching Hartmann number $M$. As expected, velocity reduces effectively for $M$ which can be justifies due to role of Lorentz force. Figure 1c predicts that $f^{\prime}$ increases for enlarging variable viscosity constant $\gamma_{1}$. Figure 3a aims to express the change in temperature $\theta$ when $\beta$ is maximum. The upshot behavior of temperature has been noted for enlarging $\beta$. The physical interpretation of variable thermal conductivity constant $\varepsilon$ on $\theta$ is predicted in Fig. 3b. The thermal profile gets more maximum trend subject to increase $\varepsilon$. In fact, the consideration of variable thermal conductivity fluctuated the heat transfer phenomenon more effectively as it varies with various factors. Figure 3c imposed the role of radiation constant $Rd$ on $\theta$. The presence of radiative phenomenon incorporated more heat transfer impact and boosted the thermal pattern. The radiative phenomenon is associated to the transmission of energy in terms of electromagnetic waves. Figure 3d discusses the onset of Prandtl number $\Pr$ on $\theta$. The reduction is noted for temperature due to enlarging $\Pr$. Physically, such effects are due to controls of thermal diffusivity which preserving opposite relation with Prandtl number.

**Figure 4:**
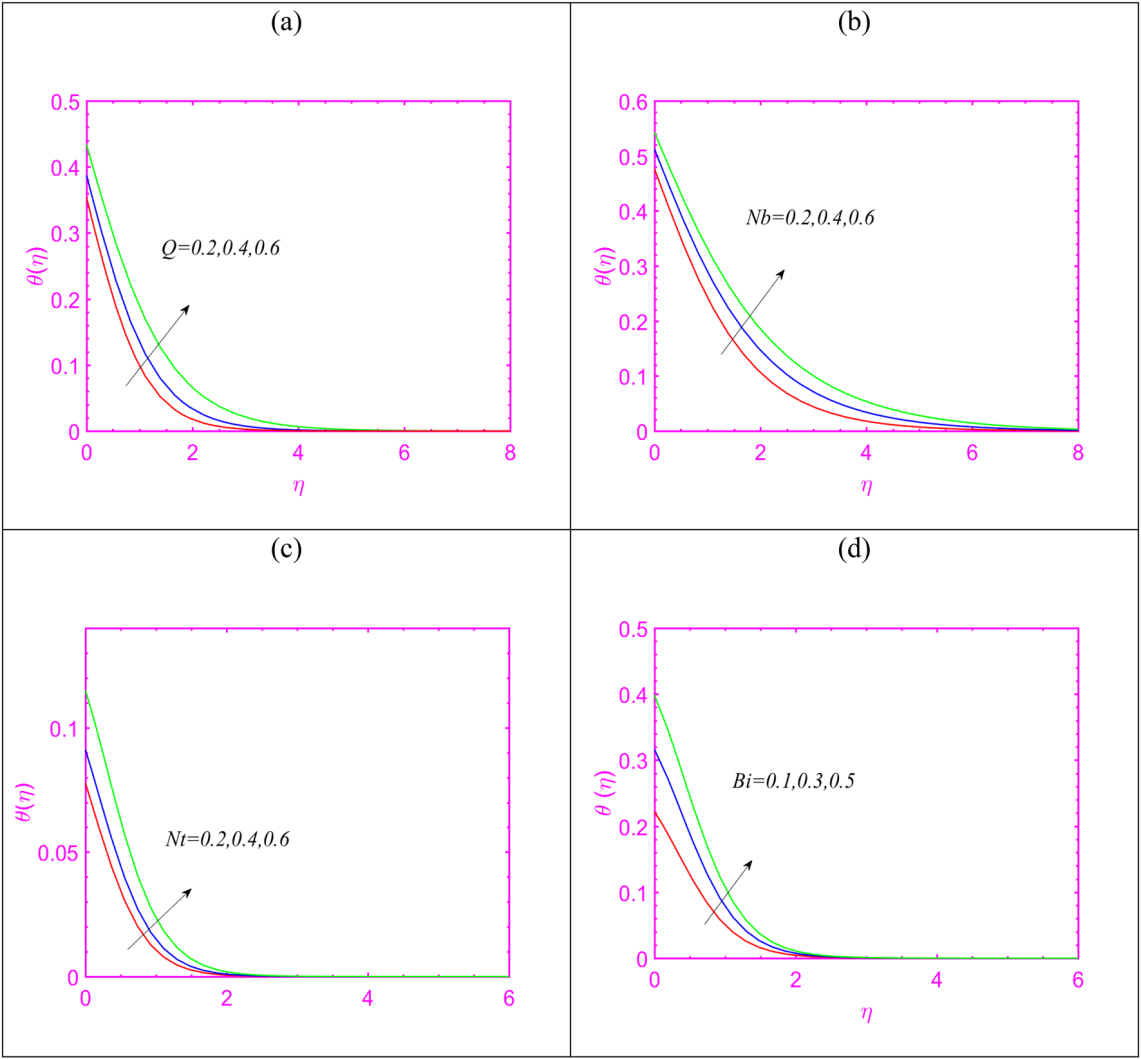
(**a**–**d**) Assessment of temperature for (**a**) $Q$, (**b**) $Nb$, (**c**) $Nt$ and (**d**) $Bi.$

In order to evaluate the characteristics of heat source $Q$ on $\theta$, Fig. 4a has been presented. Physically, the presence of $Q$ identifying some external heat source which can increases the heat transfer determination. In order to discuss the contribution of $Nb$ and $Nt$ on $\theta$, Fig. 4b,c are plotted, respectively. First, increasing outcomes in heat transfer fluctuation are noted due to both $Nb$ and $Nt$. The increasing in heating capacitance due to $Nb$ is justified as nanoparticles specifies random motion due to which increment in $\theta$ is noted. On other hand, the thermophoresis parameter justifies the migration of fluid in relatively cooler regime which results in increment in temperature. The effects of Biot number $Bi$ on $\theta$ is examined in Fig. 4d. The enhancing change is noticed for $\theta$ with increasing $Bi$. Physically, the Biot number is associated to the heat transfer coefficient which enriches the heat transfer impact.

**Figure 5:**
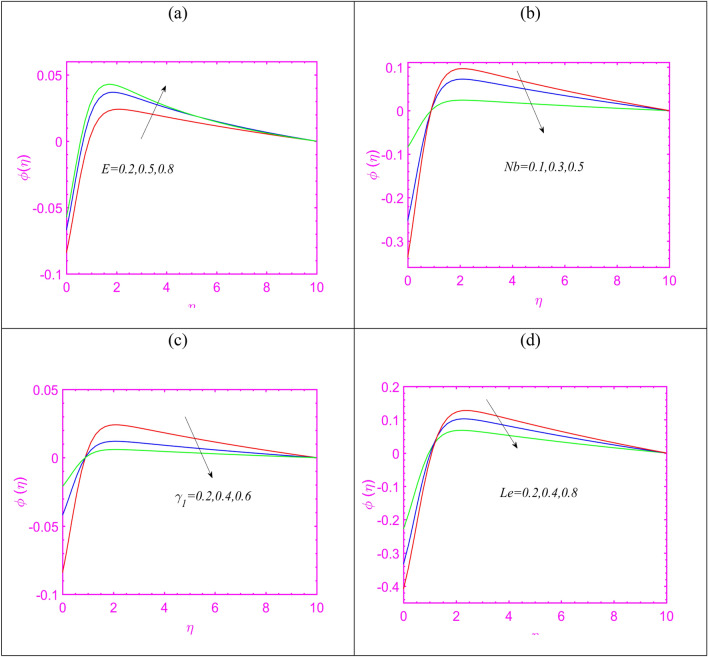
(**a**–**d**) Assessment of temperature for (**a**) $E$, (**b**) $Nb$, (**c**) $\gamma_{1}$ (**d**) $Le.$

**Figure 6:**
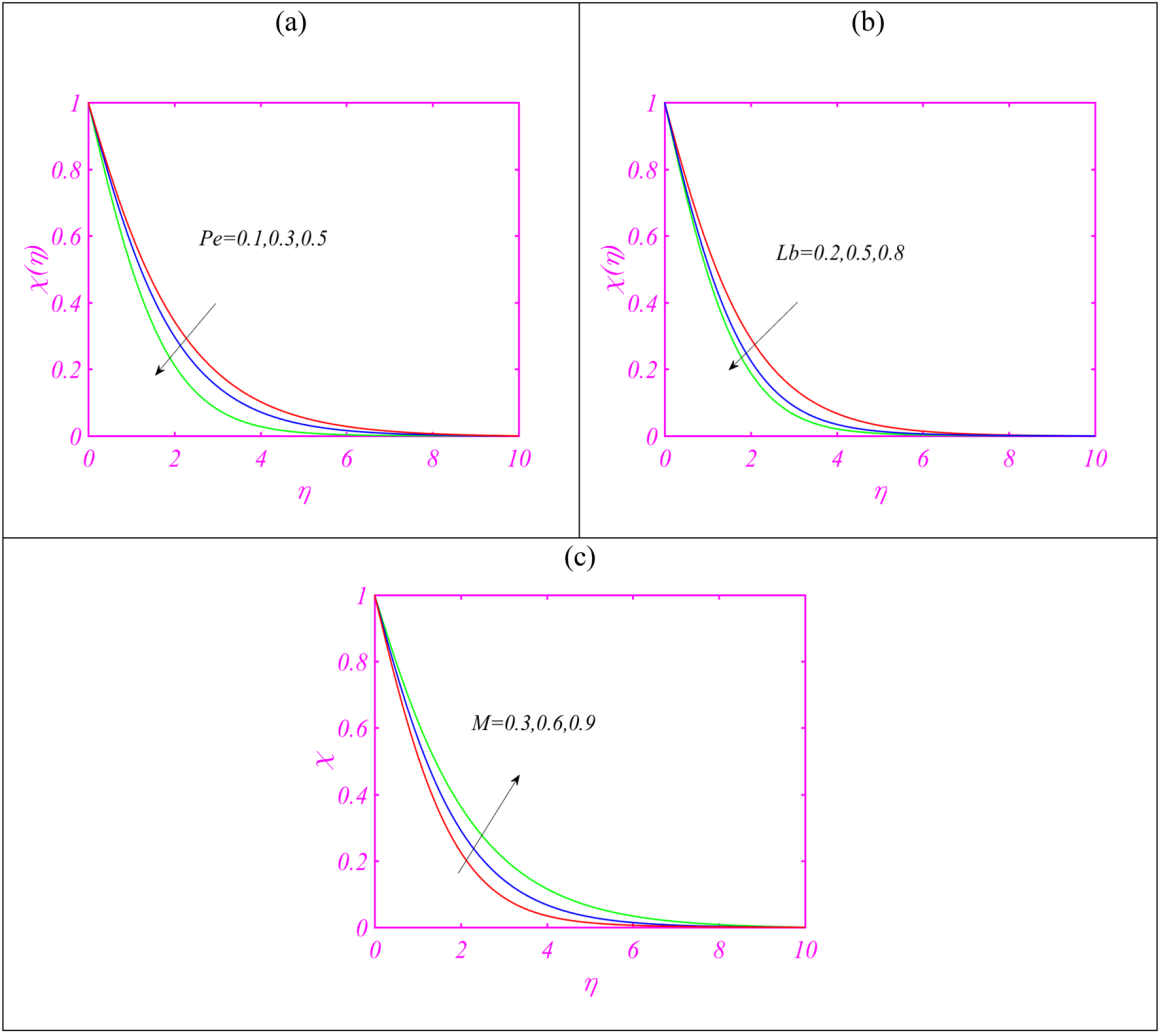
(**a**–**c**) Assessment of microorganism profile for (**a**) $Pe$, (**b**) $Lb$ (**c**) $M.$

Figure 5a presents the concentration profile $\phi$ in view of leading aspects of activation energy constant $E$. The concentration profile enriches when the effects of activation energy are utilized. The activation energy is important in initiating the chemical reaction process. Figure 5b justifying the importance of Brownian constant $Nb$ on $\phi$. Here, the declining rate of concentration is exhibited for $Nb$. Figure 5c demonstrates that $\phi$ reduces with $\gamma_{1}$. The graphical effectors made in Fig. 5d reporting the insight of $\phi$ due to enriching values of Lewis number $Le.$ The Lewis number expresses contrasting relation with mass diffusivity which turning down concentration phenomenon. Figure 6a is presented in order to judge the influence of Peclet number $Pe$ on microorganisms profile $\chi$. Lower observations are scaled out for $\chi$ in view of increasing $Pe$. Physical aspects behind such change is smaller motile density. Same results are examined in Fig. 6b in case of increasing $Lb.$ Figure 6c claims that $\chi$ boosted for increasing $M.$

Table 2 identifies the numerical calculation of wall shear force under the variation of emerging parameters. Larger variation in $- f^{\prime\prime}\left( 0 \right)$ is observed for $\beta$. However, decreasing aspect of $- f^{\prime\prime}\left( 0 \right)$ is reported for $\gamma_{1}$. Table 3 presents change in $- \theta^{\prime}\left( 0 \right)$ due to different constants. It is noted that $- \theta^{\prime}\left( 0 \right)$ declined for $\varepsilon$, $Q$ and $Nb$. From Table 4, it is examined that $- \phi^{\prime}\left( 0 \right)$ gets maximum variation for $Le$ while less results are predicted for $\beta$. Table 5 claims an increasing results for $- \chi^{\prime}\left( 0 \right)$ against $Pe$ and $Lb.$

**Table 2:** Numerical calculation of $- f^{\prime\prime}(0)$.

$M$	$\beta$	$\gamma_{1}$	$- f^{\prime\prime}\left( 0 \right)$
0.2	0.1	0.1	0.589053
0.4			0.645329
0.6			0.690325
	0.4		0.7874201
	0.8		0.842655
	1.2		0.8948755
		0.1	0.924547
		0.3	0.914624
		0.5	0.854766

**Table 3:** Numerical calculation of Nusselt number.

$\varepsilon$	$Rd$	$\Pr$	$Q$	$Nb$	$- \theta^{\prime}\left( 0 \right)$
0.1					0.352164
0.3					0.284876
0.5					0.2446830
	0.2				0.365648
	0.4				0.454648
	0.6				0.581325
		0.3			0.458895
		0.7			0.614654
		0.9			0.98322
			0.2		0.484565
			0.4		0.436889
			0.8		0.41598
				0.2	0.513265
				0.4	0.487798
				0.7	0.435488

**Table 4:** Numerical calculation of Sherwood number.

$\beta$	$Nt$	$Le$	$- \varphi^{\prime}\left( 0 \right)$
0.2	0.1	0.3	0.3165658
0.4			0.2548766
0.6			0.184565
0.1	0.1		0.3579889
	0.3		0.3423215
	0.5		0.297988
		0.2	0.3598988
		0.6	0.4523565
		0.8	0.4987820

**Table 5:** Numerical calculation of motile density number.

$Pe$	$Lb$	$- \chi^{\prime}\left( 0 \right)$
0.1	0.5	0.5145465
0.3		0.5698899
0.7		0.6245565
0.2	0.2	0.5045666
	0.4	0.5854798
	0.6	0.648778

## Conclusions

The bioconvective flow of Maxwell nanofluid under the assumptions of variable thermal conductivity and slip effects have been studied. The novel contribution of radiative phenomenon and activation energy is endorsed. The shooting technique is utilized for solving to problem. Major results are:

- A reduction in velocity profile is noticed due to relaxation time constant.
- An increment in velocity is exhibited due to variable viscosity constant.
- The variable thermal conductivity parameter enhanced the temperature profile more effectively s compared to constant assumptions of thermal conductivity.
- The temperature profile enhanced due to relaxation constant.
- By increasing Biot number and external heat source, the heat transfer phenomenon boosted.
- The microorganisms profile enhanced with Hartmann constant.
- These results can be further updated by utilizing the cubic autocatalysis chemical reaction, thermos-diffusion effects and Joule heating^30,31^.

## Data Availability

The datasets used and/or analyzed during the current study available from the corresponding author on reasonable request.

## List of symbols

$$u,v$$: Velocity components
$$T$$: Temperature
$$T_{\infty }$$: Ambient temperature
$$C$$: Concentration
$$\rho_{f}$$: Fluid density
$B_{0}$: Magnetic field strength
$\mathop K\limits^{ \simeq } (T)$: Variable thermal conductivity
$$D_{B}$$: Brownian diffusion
$k^{ * }$: Mean absorption coefficient
$Q_{0}$: Heat source coefficient
$\Pr$: The Prandtl number
$R_{d}$: The radiation parameter
$\beta$: The Maxwell parameter
$\varepsilon$: The Curie temperature parameter
$N_{t}$: The thermophoresis parameter
$$Pe$$: Peclet number
$Nu_{x}$: Local Nusselt number
$Sh_{x}$: Local Sherwood number
$$T_{w}$$: Surface temperature
$$C_{\infty }$$: Ambient concentration
$$\lambda$$: Relaxation time
$\mathop \mu \limits^{ \simeq } (T)$: Temperature dependent viscosity
$$\sigma$$: Electrical conductivity
$$\rho c_{p}$$: Specific heat
$$q_{r}$$: Radiative flux
$$\hat{b}$$: Chemotaxis constant
$Re_{x}$: Local Reynolds number
$D_{m}$: Microorganisms diffusion constant
$\gamma_{1}$: The Biot number
$Le$: The Lewis number
$$M$$: Hartmann parameter
$N_{b}$: The Brownian motion parameter
$Q$: The heat source /sink parameter
$\delta_{1}$: Microorganism difference parameter
$C_{fx}$: Skin friction coefficient
$Nn_{x}$: Motile density number
